# Overnight total thyroidectomy: a safe management

**DOI:** 10.1007/s00423-025-03918-y

**Published:** 2025-12-25

**Authors:** Riccardo Morandi, Claudio Guarneri, Matteo Nardin, Stefania Maria Filomena Mitola, Eleonora Valloncini, Elisa Gatta, Pietro Bellini, Francesco Bertagna, Carlo Cappelli, Claudio Casella

**Affiliations:** 1 Department of Clinical and Experimental Sciences, Surgical Clinic, University of Brescia, ASST Spedali Civili, Brescia, Italy; 2 General Surgery, ASST Spedali Civili Di Brescia PO Montichiari, Brescia, Montichiari, Italy; 3 Internal Medicine, Department of Medicine, ASST Spedali Civili, Brescia, Italy; 4 Department of Molecular and Translational Medicine, University of Brescia, Brescia, Italy; 5 Department of Clinical and Experimental Sciences, SSD Endocrinologia, University of Brescia, ASST Spedali Civili, Brescia, Italy; 6 Centro per la Diagnosi e Cura delle Neoplasie Endocrine e delle Malattie della Tiroide, University of Brescia, Brescia, Italy; 7 Nuclear Medicine, Department of Medical and Surgical Specialties, Radiological Sciences and Public Health, ASST Spedali Civili Di Brescia, University of Brescia, Brescia, Italy

**Keywords:** Overnight thyroidectomy; selection criteria, Postoperative complication, Early discharge, Total thyroidectomy

## Abstract

**Introduction:**

Rapid discharge protocols have gained progressive popularity even in thyroid surgery due to the superimposable risks of complications compared to inpatient management and for the subsequent increased surgical volume. The aim of this study is to evaluate results, benefits and complications’ rates of a rapid discharge in postoperative day 1 (POD1 - overnight thyroidectomy) among patients submitted to total thyroidectomy at our surgical clinic.

**Materials and methods:**

Single centre retrospective analysis of 729 patients submitted to total thyroidectomy between 2016 and 2024; 402 patients who are scheduled for discharge on POD 1 and 327 patients discharged after a minimum of 72 hours observation (POD 3). Data concerning postoperative complications (POC) at 24 hours, 10 and 30 days were collected. Patients’ satisfaction about the rapid discharge protocol was also registered.

**Results:**

We registered no significative differences between incidence of complications at 24h, 10-days or 30-days re-evaluations in POD1 and POD3 groups. Graves’ Disease represents the main context in which early postoperative (24h) and overall complications occurred. The 94.6% of POD1 patients reported a global satisfaction in the rapid discharge scenario.

**Conclusions:**

POD1 patients are not exposed to additional postoperative risk with overnight thyroidectomy following total thyroidectomy, given accurate patient selection. Early and overall complications are more frequently observed in patients with Graves' disease. Overnight thyroidectomy, combined with thorough perioperative patient education, received widespread appreciation among our surgical cohort.

## Introduction

 Thyroidectomy is a common surgical intervention indicated for both neoplastic and benign pathologies, representing a frequent cause of hospitalization in neck surgical practice. It is estimated that approximately 40,000 thyroidectomies are performed annually in Italy [1] with an increasing trend over the past decades due to historically high geographic incidence of thyroid disease and a higher rate of cancer diagnosis [2].

Thyroidectomy is typically performed as an elective procedure, but urgent thyroidectomies may be performed in cases of tracheal stenosis, haemorrhages, or thyrotoxic states [3, 4].

Over the past decade, thyroid surgery has undergone significant changes with the advent of transoral, transaxillary, and endoscopic thyroidectomy techniques. Concurrently, hospital stays have been reduced from two to three days to same-day discharge (outpatient thyroidectomy) or next-day discharge protocols (overnight thyroidectomy), which have become increasingly common [5, 6].

According to Literature, selection criteria for rapid discharge include favourable preoperative conditions concerning comorbidities, drug therapies, social support, and geographical context to mitigate postoperative complications [7].

Several high-volume studies have validated the safety of rapid discharge protocols, showing no additional risks in managing postoperative complications as outpatients [8–10] whilst some other Authors claim that outpatients setting could cause delays in management in case of acute complications due to distance, traffic or transport issues [11].

Main surgical complications include bleeding, recurrent laryngeal nerve (RLN) injuries, hypoparathyroidism with hypocalcemia, and wound complications (infections, seromas, hematomas) necessitating rapid detection and treatment [12–14].

The COVID-19 pandemic prompted the strengthening of hospitalization management protocols, including in ordinary surgical practice.

In March 2021, a dedicated surgical division was established in our hospital to facilitate ambulatory and overnight surgical procedures. The success of this division relies on rapid patient turnover without compromising safety and quality of care.

The shortened observation period required for overnight thyroidectomy compared to inpatient procedures necessitates careful patient selection. According to Literature, common exclusion criteria encompass cervical lateral/distant lymph node metastasis, invasion of surrounding tissues by the thyroid gland, American Society of Anesthesiology (ASA) classification of grade III or higher, significant chronic conditions, including severe liver or kidney impairment (chronic kidney disease - CKD - stage III-IV-V), and current use of anticoagulant or antiplatelet medications [7].

Despite the safety of these protocols, the potential for complications can deter both clinicians and patients from opting for rapid discharge [15].

This study aims to evaluate the outcomes of rapid discharge on postoperative day 1 (POD1 - overnight thyroidectomy) among patients undergoing total thyroidectomy at our surgical clinic.

## Materials and methods

### Study population

In the study design, two cohorts with superimposable characteristics were defined: one consisting of patients treated with total thyroidectomy and discharged after a minimum of 72 h (POD3) up until 2021, and the other comprising patients treated with total thyroidectomy after 2021 and eligible for discharge on postoperative day 1 (POD1).

We included all patients treated with conventional cervicotomic total thyroidectomy between January 2016 and March 2024 at General Surgery department of the ASST Spedali Civili Brescia, Italy.

Surgical indications included multiglandular goitres, Graves’ disease, or nodular goitres requiring total thyroidectomy, according to the 2015 American Thyroid Association (ATA) guidelines.

All thyroidectomies were performed by the same surgical team with over twenty years of experience in thyroid and parathyroid surgery, no differences in surgical technique were applied during the period taken into consideration.

Exclusion criteria were a history of neck surgery, head/neck cancer, preoperative suspicion of laterocervical nodal pathology, local radiation therapy (RT), cervical-mediastinal goiters requiring sternotomic access, CKD above stage II, Body Mass Index (BMI) greater than 35 kg/m^2^ and ASA class 3–4 patients, who required a planned mean hospital stay of 3 days.

Patients, matched for baseline characteristics and comorbidities, were considered eligible for this retrospective single-institution study.

All patients received comprehensive postoperative education, signed informed consent forms, and were given detailed informational materials.

Emergency contact details were also provided to ensure prompt communication with dedicated medical staff.

### Discharge protocols

According to previous discharge protocol active at our department, patients treated before 2021 were discharged after a minimum of 3 days of observation.

According to Literature eligibility criteria for rapid discharge protocols [16], starting from 2021 all eligible patients were scheduled for discharge on postoperative day 1.

Patients with insufficient social support or poor logistical independence were excluded from the rapid discharge protocol to ensure adequate access to urgent re-evaluation.

Hemithyroidectomies and adjunctive parathyroid explorations were not considered in this study.

Postoperative care strategy included routine administration of hormone replacement therapy, oral calcium and vitamin D supplements in all patients.

Collegial re-evaluation with endocrinologist was then performed to adjust the hormone replacement therapy and to define the correct timing of calcium supplementations.

### Endpoints

Data on postoperative complications (POC) at 24 h, 10 days (from day 2 to day 10), and 30 days (from day 11 to day 30) were collected.

In order to detect postoperative hypocalcemia, plasma calcium level was measured at 24 h, 10 days, and 30 days after surgery.

Similarly, in order to find out hypoparathyroidism parathormone levels were measured at 10 days, and 30 days.

Acute bleeding (clinically or radiologically detected), RLN palsy (confirmed by direct fibro laryngoscopy performed in all patients before discharge), hypocalcemia (PTH < 15 pg/mL) hypocalcemia (ionized plasma calcium concentration < 4.53 mg/dL with or without symptoms), and wound complications (infection – seroma – superficial hematoma) were clinically identified and collected as postoperative complications of interest.

Patient satisfaction with the rapid discharge protocol was assessed through oral interviews during the 30-day follow-up evaluation.

### Statistical analyses

Data analysis and management were performed using IBM^®^ SPSS^®^ Statistics 20 for Windows^®^ software. A probability value of *p* < 0.05 was considered statistically significant. The Shapiro-Wilk test was used to assess normality. Continuous variables were expressed as mean values ± standard deviation (SD) or median and interquartile range (IQR) as appropriate, and categorical variables were expressed as numbers (percentage). Fisher’s exact test was used for categorical data comparisons, and the Mann–Whitney U test was used to determine differences between groups.

## Results

**Fig. 1:**
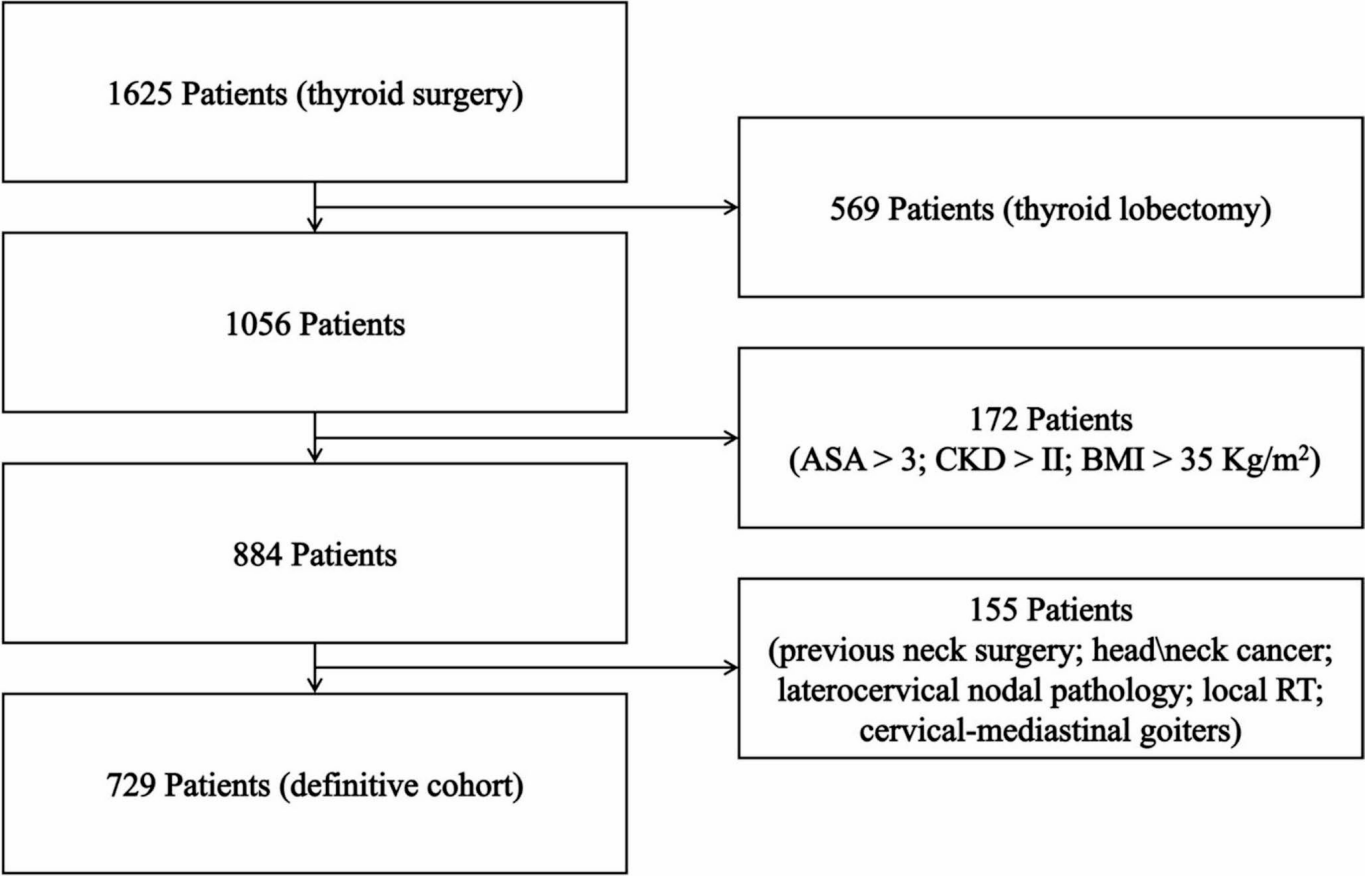
Study Selection Flow diagram. ASA: American Society of Anesthesiology; CKD: chronic kidney disease; BMI: body mass index; RT: radiation therapy

The flow diagram (Fig. 1) illustrates all the patients, matched for baseline characteristics and comorbidities, who were considered eligible for this retrospective single-institution study.

A total of 729 patients were submitted to total thyroidectomy; 402 patients composed the POD1 group, while 327 patients were enrolled in POD3 group.

The global mean age was 48.51 ± 14.52 y.o. and no significative differences (*p* = 0.732) were found between POD1 (49.01 ± 14.87 y.o.) and POD3 (47.81 ± 14.24 y.o.). Main baseline characteristics were superimposable between the two groups, as shown in Table 1.

**Table 1:** Cohorts demographic’s characteristics

	POD 1 (*n* = 402)	POD 3 (*n* = 327)	*p*-value *
Age (years)	50.9 (14.0)	53.0 (13.1)	0.253
Male	73 (18.2)	51 (15.6)	0.340
Female	329 (81.8)	276 (84.4)	0.414
Hypertension	85 (21.1)	68 (20.8)	0.960
Diabetes Mellitus	29 (7.2)	15 (4.6)	0.150
History of Heart Failure	17 (4.2)	12 (3.7)	0.430
COPD	23 (5.7)	14 (4.3)	0.450
OSAS	55 (13.7)	33 (10.1)	0.290
Asthma	25 (6.2)	13 (4.0)	0.160
Chronic Kidney Disease (Stage I, II)	34 (8.5)	17 (5.2)	0.100
First degree obesity (BMI 30–35 kg/m^2^)	47 (11.7)	33 (10.1)	0.810
Smokers	84 (20.9)	55 (16.8)	0.760

Data collection: Mean (IQR) or *n* (%). *Fisher's Exact Test or Mann-Whitney U test were used

*BMI* = body mass index, *COPD* = chronic obstructive pulmonary disease, *OSAS* = obstructive sleep apnea syndrome, *POD1* = postoperative day1 cohort, *POD3* = postoperative day 3 cohort

A total of 470 (64.4%) patients affected by Graves’ Disease (GD), 90 (12.4%) patients affected by uninodular goitre, and 169 (23.2%) cases of multinodular goitre were included in this surgical series.

Analysing the incidence of main postoperative complications we did not find any significative difference between the two groups (Tables 2, 3 and 4).

**Table 2:** 24 h postoperative complications’ incidence in POD1 and POD3 cohorts

24 h POC	POD1	POD3	*p*-value *
Bleedings	4 (0.9%)	3 (0.9%)	0.593
Hypocalcemia	37 (9.2%)	35 (10.7%)	0.272
RLN palsy	29 (7.2%)	32 (9.8%)	0.593

Data collection: *n* (%). *Fisher’s Exact Test or Chi-squared test (χ² test) were used.

POD1: Postoperative day1 cohort. POD3: Postoperative day 3 cohort.

**Table 3:** 10-Days postoperative complications’ incidence in POD1 and POD3 cohorts

10-Days POC	POD1	POD3	*p*-value *
Hypoparathyroidism	8 (2.0%)	6 (1.8%)	1.000
Wound’s infection	12 (3.06%)	9 (2.8%)	1.000

Data collection: *n* (%). *Fisher’s Exact Test or Chi-squared test (χ² test) were used

POD1: Postoperative day1 cohort. POD3: Postoperative day 3 cohort

**Table 4:** 30-Days postoperative complications’ incidence in POD1 and POD3 cohorts

30-Days POC	POD1	POD3	*p*-value *
Hypoparathyroidism	4 (1.0%)	3 (0.9%)	1.000
Wound’s infection	4 (1.0%)	3 (0.9%)	1.000

Data collection: *n* (%). *Fisher’s Exact Test or Chi-squared test (χ² test) were used

POD1: Postoperative day1 cohort. POD3: Postoperative day 3 cohort

In Table 2 complications at 24 h are reported.

Acute bleeding was observed both on POD1 (4 cases, 0.9%) and in the POD3 group (3 cases, 0.9%) (*p* = 0.593). All cases were surgically managed in the operating room as urgent or emergent procedures; hypoparathyroidism occurred in 37 cases (9.2%) among POD1, while 35 cases (10.7%) were registered in POD3 with *p* = 0.272; RLN palsy was assessed in 29 patients (7.2%) in POD1 and in 32 patients (9.8%) in POD3 (*p* = 0.593).

From the analysis of the totality of 24 h POC registered in both cohorts, no significative differences were registered (*p* = 0.205).

POD1 patients who registered any of the 24 h POC (70 cases, 17.4%) continued their hospital stay to ensure adequate care.

Ten-days re-evaluation collected all complications from 48 h to 10 days after surgery.

Eight cases of hypoparathyroidism (2.0%) and 6 cases (1.8%) respectively in POD1 and in POD3 (*p* = 1.000) were found, while wound’s infection was registered in 12 (3.06%) and 9 patients (2.8%) respectively in POD1 and in POD3 (*p* = 1.000) (Table 3).

Concerning global 10-days complications, no statistical differences were assessed between the two groups (*p* = 0.945). No complications arising on the second and third postoperative days were recorded, in particular no bleedings were registered.

Thirty-days complications (from day 11 to day 30) included 4 cases of hypoparathyroidism (1.0%) in POD1 and 3 cases (0.9%) in POD3 (*p* = 1.000); wounds’ infection was found in 4 patients (1.0%) among POD1 and in 3 patients (0.9%) among POD3 (*p* = 1.000) (Table 4). No statistical p value was found to be associated to the sum of the complications (*p* = 1.000).

No re-hospitalisation was needed in management of 10 and 30-days hypoparathyroidism findings since an increased oral calcium supplementation dosage was found out to be sufficient.

In the same way, a nonoperative management with oral antibiotic therapy and outpatients’ medications was applied in all wound’s infection cases.

No cases of bleeding or RLN palsy were registered in both groups at the 10- and 30-days revaluations.

We also performed a subpopulation analysis of POC incidences with a division of the population based on the baseline pathology that brought to surgical intervention.

**Fig. 2:**
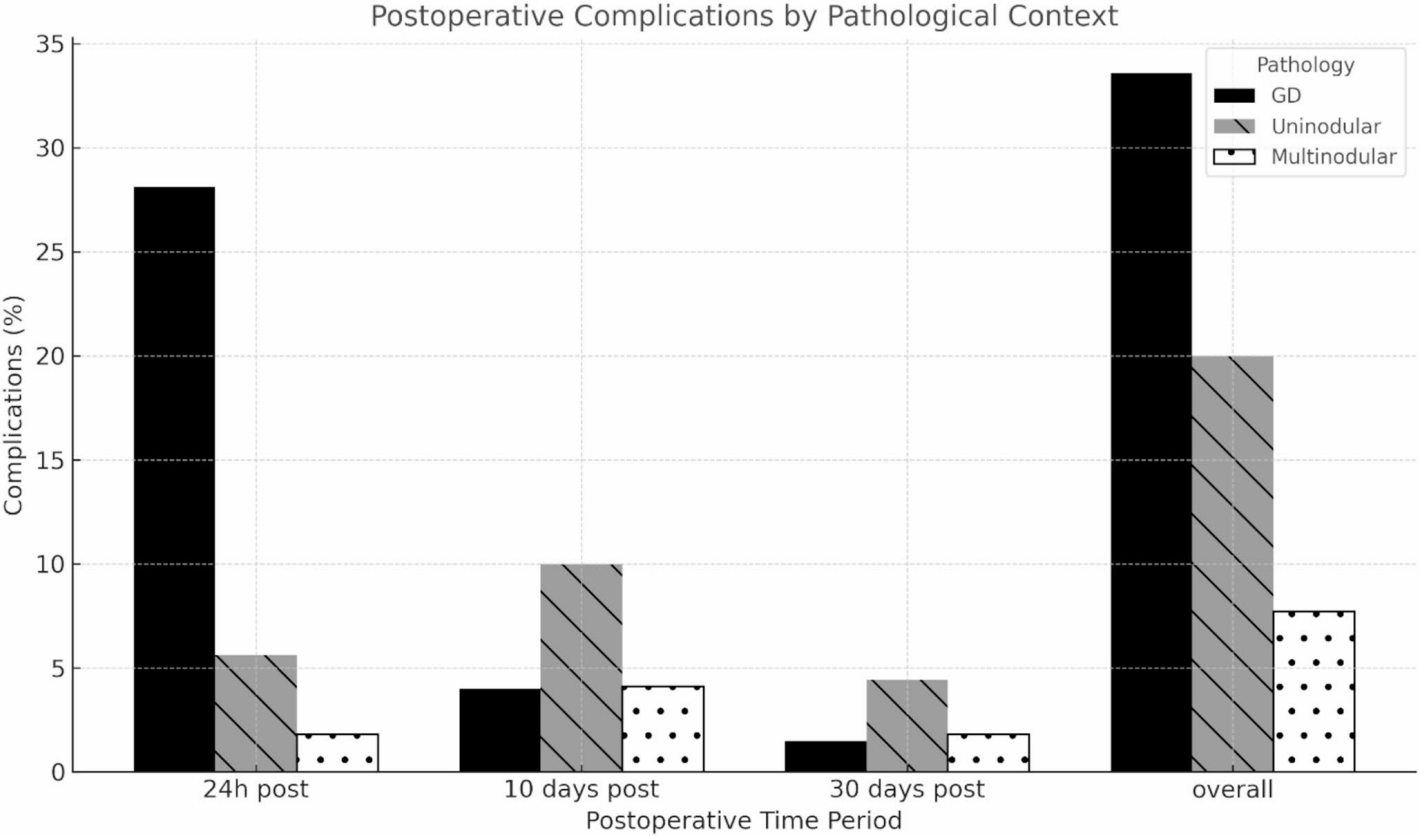
Postoperative Complications’ incidence and pathological context

As shown in Tables 5, 6, 7, and 8 and in Fig. 2, GD represents the main context in which early postoperative (24 h) and overall complications occurred, with significative p values. Uninodular goitre appears to have a higher incidence of 10 days postoperative complications (*p* = 0.048).

**Table 5:** 24 h postoperative complications’ incidence and pathological context

Disease	24 h POC	*p*-value *
GD	132 (28.1)	*P* < 0.001
Uninodular goitre	5 (5.6)	
Multinodular goitre	3 (1.8)	

Data collection: *n* (%). *Fisher’s Exact Test or Chi-squared test (χ² test) were used

POC: Postoperative Complication; GD: Graves’ Disease

**Table 6:** 10-Days postoperative complications’ incidence and pathological context

Disease	10-Days POC	*p*-value *
GD	19 (4.0)	*P* = 0.048
Uninodular goitre	9 (10.0)	
Multinodular goitre	7 (4.1)	

Data collection: *n* (%). *Fisher’s Exact Test or Chi-squared test (χ² test) were used

POC: Postoperative Complication; GD: Graves’ Disease

**Table 7:** 30-Days postoperative complications’ incidence and pathological context

Disease	30-Days POC	*p*-value *
GD	7 (1.5)	*P* = 0.171
Uninodular goitre	4 (4.4)	
Multinodular goitre	3 (1.8)	

Data collection: *n* (%). *Fisher’s Exact Test or Chi-squared test (χ² test) were used

POC: Postoperative Complication; GD: Graves’ Disease

**Table 8:** Overall postoperative complications’ incidence and pathological context

Disease	Overall POC	*p*-value *
GD	158 (33.6%)	*p* < 0.001
Uninodular goitre	18 (20%)	
Multinodular goitre	13 (7.7%)	

Data collection: *n* (%). *Fisher’s Exact Test or Chi-squared test (χ² test) were used

POC: Postoperative Complication; GD: Graves’ Disease

We also analysed the overall patients’ satisfaction in a rapid discharge experience.

From the POD1 group, 332 patients (82.6%) were effectively discharged at 24 h, and among those, 314 (94.6%) reported overall satisfaction with the rapid discharge protocol at the 30-day postoperative interview (*p* < 0.001).

## Discussion

This study confirms that early discharge after total thyroidectomy (overnight thyroidectomy) is a safe and effective approach, comparable to a prolonged 72-hour hospital stay.

No statistically significant differences were observed in the overall complication rates between patients discharged on postoperative day 1 (POD1) and those discharged on POD3.

In the first 24 h, the incidence of major complications such as postoperative bleeding, transient hypocalcemia, and recurrent laryngeal nerve palsy was low in both cohorts, with no significant differences (all p-values not significant). Importantly, all acute events were promptly recognized and adequately managed, regardless of the discharge protocol.

At the 10-day follow-up, complication rates remained low in both groups. Conditions such as transient hypoparathyroidism and minor wound infections occurred with comparable frequency, again without statistically significant differences between POD1 and POD3 (p values consistently > 0.05).

By the 30-day evaluation, late complications were rare and evenly distributed across both groups, with no evidence of delayed bleeding or persistent nerve injuries. The comparison confirmed the absence of statistically significant differences, further supporting the safety and reproducibility of the overnight protocol.

A crucial factor ensuring the safety of early discharge is a rigorous patient selection process. According to Su [10] inclusion criteria for overnight thyroidectomy include patients under 60 years of age, without lateral cervical lymph node or distant metastases, and without severe chronic diseases.

The main exclusion criteria include invasion of adjacent tissues, ASA classification III or higher, and active anticoagulant or antiplatelet therapy.

Our study aligns with these selection criteria, reinforcing the importance of careful patient evaluation to minimize postoperative risks.

A key finding from our study is the high incidence of complications among patients with Graves’ Disease, emphasizing the need for special consideration in this subgroup.

Among all complications recorded within 24 h, 28.1% occurred in patients with Graves’ Disease (*p* < 0.001), highlighting the inflammatory and vascular challenges associated with this condition during surgery [17].

This aligns with previous studies, which identify Graves’ Disease as a major risk factor for transient hypoparathyroidism [18].

Proactive calcium and vitamin D supplementation in the immediate postoperative period has been suggested as an effective strategy to mitigate this risk, as implemented in our protocol.

One of the most compelling aspects of early discharge is the high level of patient satisfaction. In our study, 94.6% of patients (314 of 332 discharged at 24 h) reported a positive experience with the early discharge protocol, which aligns with findings in Literature demonstrating that recovering in a familiar environment significantly reduces stress and postoperative anxiety [19, 20].

This patient-centred approach not only enhances satisfaction but also contributes to a more efficient healthcare system without compromising safety. However, despite these benefits, some authors suggest that patients undergoing overnight thyroidectomy may experience higher levels of stress and anxiety compared to those with prolonged hospitalization [21].

This underscores the importance of targeted psychological support strategies and enhanced patient communication prior to discharge, ensuring that patients feel confident and well-prepared for home recovery.

Currently, no universally accepted guidelines exist for selecting patients for rapid discharge after thyroidectomy. However, studies suggest that strict preoperative selection criteria, clear discharge standards, and a well-coordinated multidisciplinary team equipped with robust outpatient facilities play a determinant role in successful implementation [22].

Our findings support the adoption of structured follow-up protocols to monitor patients post-discharge, ensuring that complications are promptly identified and managed.

This study has several limitations. The retrospective design and absence of randomization may impact the comparability of results, even though we matched populations according to baseline characteristics. Additionally, the single-centre nature of the study could limit the generalizability of our findings. However, to our knowledge our study represents one of the largest single centre populations of overnight total thyroidectomy analysed and it could be able to provide strong evidence supporting the feasibility and safety of a rapid and efficient thyroidectomy discharge protocol.

Furthermore, only overnight thyroidectomy was analysed in this cohort, with no outpatients included. The absence of large series of total thyroidectomies managed with outpatient protocols, along with the lack of clear evidence in the Literature regarding the safety of this approach to date, combined with the geographic extension of our region, led to the decision to exclude this type of discharge from our study design.

## Conclusions

Our findings strongly support that overnight thyroidectomy is a safe and effective approach, meeting both clinical and patient-centred goals. With no increase in postoperative risks, this protocol allows patients to recover comfortably at home while maintaining stringent safety standards. The high satisfaction rate highlights a positive patient experience, reflecting the benefits of efficient healthcare delivery without sacrificing quality. This study reaffirms that, with careful patient selection and structured follow-up, overnight thyroidectomy can optimize surgical practice, enhancing both hospital efficiency and patient satisfaction in modern healthcare settings.

## Data Availability

No datasets were generated or analysed during the current study.
